# Dual-enhancement and dual-tag design for SERS-based sandwich immunoassays: evaluation of a metal–metal effect in 3D architecture

**DOI:** 10.1007/s00604-021-05125-0

**Published:** 2021-12-21

**Authors:** Ewelina Wiercigroch, Pawel Swit, Agnieszka Brzozka, Łukasz Pięta, Kamilla Malek

**Affiliations:** 1 Faculty of Chemistry, Jagiellonian University in Krakow, Gronostajowa 2, 30-387 Krakow, Poland; 2 Jagiellonian Centre for Experimental Therapeutics, Jagiellonian University in Krakow, Bobrzynskiego 14, 30-348 Krakow, Poland

**Keywords:** Surface-enhanced Raman spectroscopy, Sandwich assay, Immunoassay, Dual-tag readout, Au nanoparticles, Ag nanoparticles, Dual surface enhancement

## Abstract

The design of a sandwich-type SERS immunoassay (surface-enhanced Raman spectroscopy) is demonstrated operating in dual surface enhancement and dual-tag paradigm. The capture and detection antibodies are linked to two SERS-active substrates and form together the three-dimensional (3D) structure after specific binding to interleukin 6. A variety of metal combinations is tested (Au–Ag, Au–Au, and Ag–Ag), but an enhanced electromagnetic field is generated only due to coupling of Ag and Au nanoparticles with an Au hexagonal nanoarray. The amplified in that way Raman signals improve the limit of detection over 3 times in comparison to the assay with only one SERS-active substrate. It is also shown that the proper readout of the true-positive signal can be achieved in assays with two Raman tags, and this approach also improves LOD. For the optimal combination of the metal–metal junction and Raman tags, a linear relationship between the Raman signal and the concentration of IL-6 is obtained in the range 0–1000 pg⋅mL^−1^with LOD of 25.2 pg mL^−1^and RSD < 10%. The presented proof-of-concept of the SERS immunoassay with the dual-enhancement and dual-tag opens additional opportunities for engineering reliable SERS biosensing.

**Graphical abstract:**

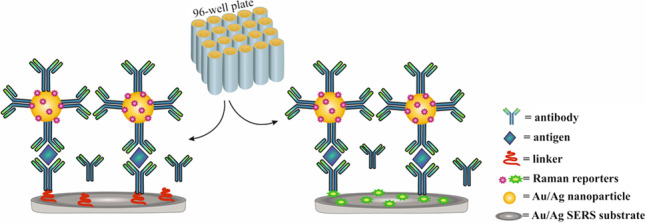

**Supplementary Information:**

The online version contains supplementary material available at 10.1007/s00604-021-05125-0.

## Introduction

Rapid and sensitive detection of biomarkers in biofluids is critical in early diagnosis as it drastically increases the treatment success. An immunoassay is one of the most common method for the quantitative analysis of biochemical targets such as proteins, hormones, or drugs, and because of that, it has been routinely employed in many areas of clinical diagnostics and life science research [1–3]. In past years, coupling of the immunoassay with various detection systems has been developed. Among the most established approaches are radiation, fluorescence, and chemiluminescence; however, enzymes are now used more frequently than any other types of labels [2–5]. Principally, the immunoassay is the bioanalytical test that measures the presence or the concentration of analytes through the specific antigen–antibody reaction. At least one reagent which is usually an antibody specific to the antigen selectively captures the analyte and/or generates a detection signal [1, 2]. The most widely used immunoassay configuration is enzyme-linked immunosorbent assay (ELISA) with the most powerful format called a “sandwich” assay. In this combination, the analyte is “sandwiched” between the capture and the detection antibodies conjugated with an enzyme. The generated signal is proportional to the amount of the analyte and its sensitivity reaches a few pg⋅mL^−1^ [1, 2, 6].

Recently, surface-enhanced Raman scattering (SERS) is increasingly considered as an ultrasensitive and rapid assay readout in the antibody-based sandwich assay [5]. SERS offers many unique advantages over conventional immunoassay based on fluorescence, electrochemistry, or ELISA, and it is believed to be a potential alternative to those methods [4, 7, 8]. The Raman scattering process is less susceptible to photobleaching due to the high stability of Raman reporters and generates narrow spectral bands suitable for multiplex detection of several biomarkers simultaneously [7, 9, 10]. In addition, the use of gold nanoparticles (Au NPs) giving surface enhancement in the red region of electromagnetic radiation minimises possible interferences from native fluorescence [4, 7, 9]. In general, the typical SERS-based immunoassay platform requires two key components—a capture substrate and a SERS immunoprobe. The capture substrate based on an antibody bonded to a solid surface (e.g. glass, Au film) is employed to extract the analyte from solution while the function of the SERS probes is the recognition of the molecule on the capture substrate and the generation of the strong SERS signal for the quantitative detection. The SERS probes are built of metal nanoparticles with good enhancement properties, labelled with a Raman reporter, and conjugated with a detection antibody [5, 9–11]. The presence and quantification of the antigen is determined based on the specific SERS spectrum of the Raman reporter since the SERS probe will not bind to the capture substrate in the absence of the target biomarker. A proof-of-concept of the immunoassays coupled with SERS was shown for the detection of cancer markers [9, 12, 13], bacterial antigen [14], and viral pathogens [15]. In these studies, the use of the Au film as the capture substrate yielded to the ng/mL sensitivity and depended primarily on the detected analyte. Some reports showed that introducing SERS-active capture substrate provides additional enhancement and improves detection sensitivity as SERS hot spots are present at the junction of two metal structures. This strategy of dual-enhancement SERS immunoassay was applied to quantify immunoglobulins [11, 16, 17], virus antigens [18], vascular endothelial growth factor (VEGF) [10], and cytokines [19] reducing the LOD from few to few ng mL^-1^. However, when the SERS probe is bonded non-specifically to the substrate, it gives rise to a false-positive signal. This issue was raised by Chuong and co-workers who designed a sandwich SERS assay using two Raman tags conjugated with AuNP and monolayered Au film [20]. In that way, true-positive signals can be separated from false readout because there are three sources of the SERS spectrum from the detection tag. False-positive signals can appear when the aggregation of AuNP and defects on the capture substrate generate strong SERS of one Raman reporter but without specific binding of the protein. Whilst bands of both Raman reporters are present in the spectrum, this signal can result only from specific binding between both antibodies and antigen (true-positives*).* This approach detected human α-thrombin with LOD of 86 pM (3.2 ng mL^-1^) [20].

In this work, to advance the development of dual-SERS tag sandwich assays, we combined SERS-active metal nanoparticles with SERS-active nanostructured substrates to provide additional enhancement of the Raman signal and consequently to improve the analytical performance of the SERS-based immunoassays. We tested them to target interleukin 6 (IL-6) which is a pleiotropic cytokine participating in normal functions of the immune system, haematopoiesis and metabolism [21]. Its circulating concentration is approximately 1 pg mL^-1^ and dramatically rises to a level of pg µg per mL under chronic inflammation and autoimmunity specific for metabolic and cardiovascular diseases, viral and bacterial infection, and sepsis [22]. The efficiency of the assays was compared to traditional designs based on the quantification of the antigen from labelled metal NPs only. We also evaluated the effect of the metal type on the operational mode of the SERS assays by using Ag and Au nanospheres and hexagonal arrays of these metals. In this way, we systematically investigated various combinations of the sandwich SERS assay to compare their efficacy and the analytical parameters. As the proposed finally assays are based on the dual-tag reporter and bimetallic system spaced by the captured protein, we expected that both tag molecules will be additionally enhanced, and the true-positive signal will be distinguished from false-positives.

## Material and methods

### Reagents

Silver and gold nanoparticles (AgNP and AuNP, respectively) of a 60-nm diameter were purchased from Ted Pella (www.tedpella.com) (USA/Canada). Non-active SERS capture substrate was prepared by sputtering a 5-nm Au film on a silica window. Reagents for the preparation of SERS-active honey-comb metal substrates, i.e. sulphuric acid (H_2_SO_4,_ 98%, p.a.), phosphoric acid (H_3_PO_4_, 85%, p.a.) and chromium trioxide (CrO_3_, p. a) were obtained from Chempur (www.chempur.pl) (Poland), while perchloric acid (HClO_4_, 60%, p.a.) was purchased in VWR Chemicals (www.pl.vwr.com) (Poland). Ag and Au sputtering were purchased from Mennica Metale Szlachetne (www.mennicametale.com.pl) (Poland).

The following reagents were used for preparation of sandwich immunoSERS assays: 4-(2-hydroxyethyl)piperazine-1-ethanesulfonic acid (HEPES; BioPerformance Certified, ≥ 99.5%, cell-cultured tested, Sigma-Aldrich (www.sigmaaldrich.com), Poland), Tween-20 (viscous liquid for molecular biology; Sigma-Aldrich (www.sigmaaldrich.com), Poland), sodium hydroxide (1.0 M NaOH for HPCE; Honeywell Fluka™ (lab.honeywell.com/en/fluka), Poland), bovine serum albumin (BSA Fraction V, pH 7.0, standard grade; Serva (www.serva.de), Poland), 4-mercaptobenzoic acid (MBA, solid 99%; Sigma-Aldrich (www.sigmaaldrich.com), Poland), 4-nitrotiophenol (NTP, solid 80%; Sigma-Aldrich (www.sigmaaldrich.com), Poland), interleukin 6 (IL-6, lyophilized recombinant mouse protein, Gibco™ (www.gibcocellcite.com, www.thermofisher.com/pl/en/home/brands/gibco), Poland), unconjugated IL-6 monoclonal antibody (Invitrogen (www.thermofisher.com/pl/en/home/brands/invitrogen), Poland), N-(3-dimethylaminopropyl)-N′-ethylcarbodiimide hydrochloride (EDC solid, 98%; Sigma-Aldrich (www.sigmaaldrich.com), Poland), N-hydroxysulfosuccinimide sodium salt (sNHS, ≥ 98%, HPLC; Sigma-Aldrich (www.sigmaaldrich.com), Poland), ɑ-mercapto-ω-amino PEG hydrochloride (PEG, Rapp Polymer (www.rapp-polymere.com), Poland), 11-mercaptoundecanoic acid (MUA, 95%; Sigma-Aldrich (www.sigmaaldrich.com), Poland), 11-mercapto-1-undecanol 99% (MU; Sigma-Aldrich (www.sigmaaldrich.com), Poland). Phosphate-buffered saline (PBS; Gibco™ (www.gibcocellcite.com, www.thermofisher.com/pl/en/home/brands/gibco), Poland) and ethyl alcohol (96%; POCH (www.poch.com.pl), Poland) were used as solvents for antibody, antigen, and washing solutions.

Ultrapure water (18.2 MΩ cm^−1^ at 25 °C) obtained from a Millipore Simplicity UV system (Millipore (www.merckmillipore.com), USA) was used for each solution. HEPES buffer at pH = 5.9 was prepared by dissolving 595.75 mg of HEPES in 50 mL of water; pH was adjusted by NaOH.

### Instrumentation

All SERS spectra were collected by using imaging mode implemented in a confocal Raman microscope (WITec Alpha 300 (www.witec.de)) equipped with a charge-coupled device (CCD) detector and a 600-groves/mm grating. Two laser excitation wavelengths at 532 nm (a laser power of 0.5 mW) and 633 nm (a laser power of 3.5 ÷ 4.0 mW) were employed. Samples were illuminated through a 40 × objective and SERS signals were acquired in a grid from a 40 μm × 40 μm area with a step size of 2.5 μm over the *x*–*y* plane (256 spectra per image, 1024 spectra per sample) and with an exposure time of 1 s. SERS images were recorded from a randomly selected area on the assay surface. The measurement time of a single image was 5 min. (in total 20 min. for a particular sandwich assay.

UV–Vis diffuse reflectance spectra were measured with the use of a UV–Vis spectrophotometer Lambda 35 (Perkin Elmer (www.perkinelmer.com)) in the spectral range of 200–1100 nm. Spectra were converted to the Kubelka–Munk function *F*(*R*) by the operation *F*(*R*) = (1 – *R*)^2^/2R, where R is a ratio of the reflectance of the sample to a reference material.

Attenuated total reflectance (ATR) Fourier transform infrared spectra (FTIR) in the range of 700–4000 cm^−1^ and with a spatial resolution of 4 cm^−1^ were acquired for functionalised nanoparticles and solid substrates. A FTIR spectrometer ALPHA model with a diamond crystal (Bruker (www.bruker.com), Germany) and an Agilent 670-IR spectrometer (www.agilent.com) combined with a 620-IR microscope were employed, respectively.

Surface morphology and chemical composition of the AAO substrate were examined by a field emission scanning electron microscope (SEM) (FE-SEM Hitachi S-4700 (www.hitachi.eu/en), Tokyo, Japan) and energy-dispersive X–ray spectroscopy (EDAX Noran System 7, Tokyo, Japan), respectively. A scanning probe image processor (WSxM 4.0 Develop 7.6) and ImageJ 1.37v software were employed to evaluate the cell diameter, pore diameter, double wall thickness, and porosity of nanostructured substrates (Fig. S1 in ESM, Electronic Supplementary Material).

### Fabrication of sandwich SERS immunoassays

**Fig. 1:**
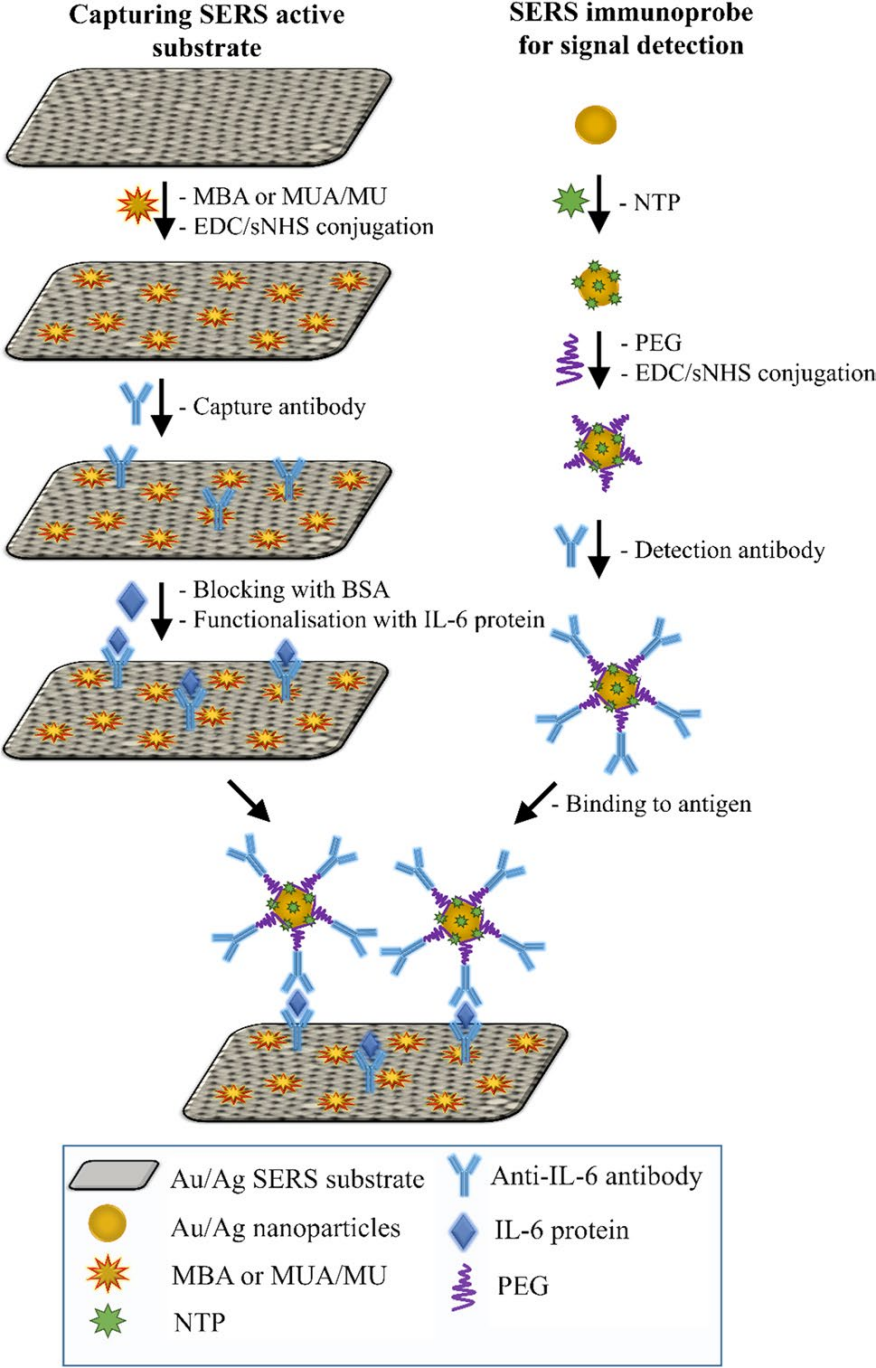
A schematic of the construction process of the sandwich SERS immunoassay

Preparation of sandwich SERS immunoassays was a two-stage process and required a separate functionalisation of nanospherical Ag and Au NPs and capture substrates. The metal solid SERS substrate was prepared according to a procedure described in detail in ESM*.* Figure 1 schematically illustrated the operating principle of the SERS immunoassay for the antigen detection.

One millilitre of Au and Ag NPs (2.6 × 10^10^ and 3.1 × 10^10^ particles⋅mL^−1^, respectively) was centrifuged and re-dispersed in HEPES buffer and then incubated with the Raman reporter—1 mM ethanolic solution of 4-nitrotiophenol (NTP). Twenty microlitres of NTP was added to 1 mL of NPs solution and kept at room temperature for 1 h on a programmable rotator. After incubation and washing with HEPES buffer, NPs solutions were incubated overnight with PEG (500 µL of 2.5 mM PEG per 1 mL of NPs) to attach the carboxyl group serving as a platform for bioconjugation. For activation of COOH groups, 20 µL of freshly prepared EDC (6 mM) and sulfoNS (15 mM) were added to a suspension of nanoparticles and stirred constantly for 25 min. An excess of EDC and sNHS was removed by centrifugation and particles were re-suspended again in HEPES buffer. Afterward, 2 µg of IL-6 antibody in 100 µL HEPES buffer was added to 1 mL of the functionalised NPs and allowed to react at room temperature for 2 h. Thirty minutes after starting incubation, 50 µL of 2% BSA/PBS solution was added to block the remaining active surface and to minimise non-specific binding. Finally, the antibody-conjugated SERS nanotags were washed twice with 2% BSA/PBS to remove non-conjugated antibodies and then were re-dispersed in 1 mL of BSA/PBS.

Au and Ag capture substrates were functionalised and activated in the following steps. After each step, all substrates were washed three times by immersion in appropriate solutions. Firstly, the substrates (with a dimension of 10 mm × 10 mm) were incubated with the second Raman reporter—1 mM ethanolic solution of 4-mercaptobenzoic acid (MBA) or with the linkers—ethanolic mixture of 100 mM 11-mercaptoundecanoic acid (MUA) and 100 mM 11-mercapto-1-undecanol (MU). For this purpose, 200 μl of solution were placed on the capture substrate for 2 h at room temperature and after that washed with EtOH:H_2_O solution (1:1) to remove an excess of MBA or MUA/MU. In the next step, the carboxyl groups of MBA and MUA were activated and stabilised by a 25-min incubation with 200 mM EDC and 50 mM sNHS. After washing with HEPES buffer, 200 µL PBS solution containing 2 µg of the capture anti IL-6 antibody was imposed on the activated substrates and left for 2 h to react. An excess of the free capture antibody was washed out with 0.05% Tween-20 in PBS. To block non-specific binding, the functionalised solid substrates were immersed in 200 µL 2% BSA/PBS solution for 1 h at room temperature and rinsed again with 0.05% Tween-20 in PBS. After that, 200 µL IL-6 antigen or PBS (as a negative control) were deposited on the substrate’s surface for a 1-h incubation and then washed out. In the final step, the functionalized metal NPs were conjugated with the functionalised capture substrates for 1 h. They were rinsed with 0.05% Tween-20 and air-dried. Of IL-6, 300 pg mL^-1^ concentration was used to evaluate the performance of the method while calibration plots were constructed for concentrations of 0, 50, 100, 300, 600, and 1000 pg mL^-1^ of IL-6 antigen.

### Data analysis

All registered SERS spectra were initially pre-processed with the use of routine cosmic ray removal (CRR) and background correction protocol implemented in a WITec Project software (ver. 5.1) (www.witec.de). The same software was employed to perform *k*-means cluster analysis (KMC) to generate classes of similar spectral profiles exhibiting the presence of MBA, NTP, MBA, and NTP together as well as pixels without SERS signals. Average spectra from all KMC classes of acquired images were then computed with standard deviation (*SD*). Calibration plots were determined by calculation of integral intensities of SERS bands for the dual-tag (NTP: 1338 and MBA: 1588 cm^−1^) and single-tag assays (NTP: 1338 and 1572 cm^−1^) using an OPUS software (ver. 7.2). Calibration plots demonstrating a linear dependence between SERS signal and IL-6 concentrations were plotted in Microsoft Excel 2016 (www.microsoft.com)*. *The limit of detection (LOD) and limit of quantification (LOQ) were calculated according to the following equations: LOD = 3.3 × s/b and LOQ = 3 × LOD, respectively, where *s* means the standard deviation of the intercept and b is a slope of a calibration plot.

## Results and discussion

### A design of the SERS sandwich assays

**Fig. 2:**
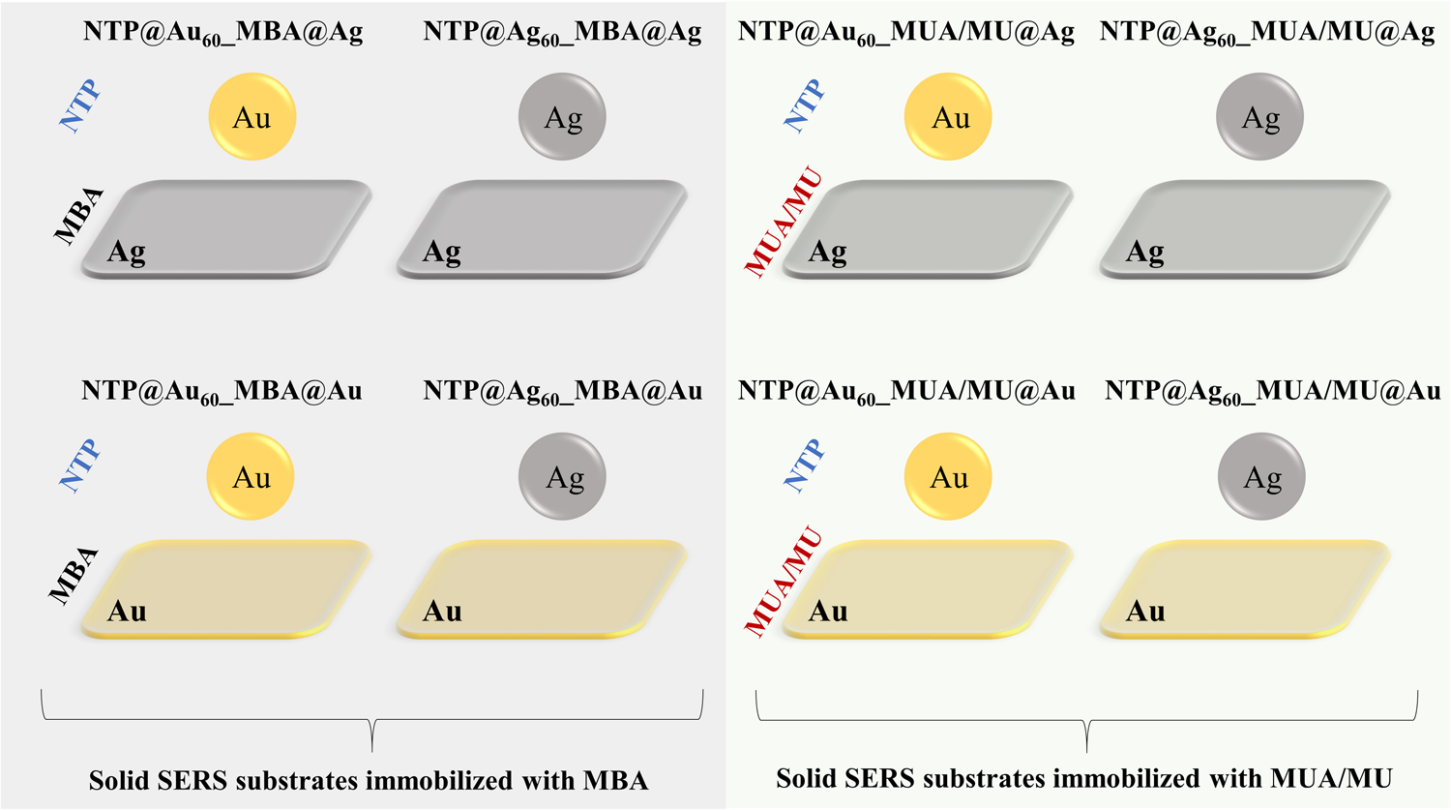
A schematic of SERS-based sandwich immunoassays prepared in various combinations of **Au** and **Ag** nanospheres with a diameter of 60 nm (**Au**_**60**_ and **Ag**_**60**_, respectively) and **Au** and **Ag** hexagonal arrays as the capture SERS-active substrates; **MBA**, **NTP**–Raman reporters; **MUA/MU**–linkers

As illustrated in Fig. 1, we constructed the sandwich immunoassays using two SERS-active substrates, i.e. commercially available gold and silver nanospheres with a diameter of 60 nm and nanostructured gold and silver solid platforms. The latter was examined by us in terms of SERS performance in the previous work [23]. Briefly, a 5 nm Au or Ag film was deposited on a nanostructured Al_2_O_3_ template which was anodized in sulphuric acid giving a closed-packed array of hexagonally arranged cells containing pores in each cell centre (Fig. S1 in ESM). Taking into account hydrodynamic radii of monoclonal antibodies and antigens (ca. 5 nm) and lengths of the linkers (PEG and MUA/MU: ca. 3.5 and 1.7 nm, respectively), we estimated that the metal NPs and the capture substrate are separated up to 20 nm apart assuming an upward orientation of the entire conjugate [24, 25]. This allowed us to achieve coherence of surface plasmon resonances between the metal NPs and nanostructured surface and to investigate an effect of coupling between Ag–Ag, Au–Au, and Ag-Au on the performance of the sandwich biosensor. In each case, silver and gold nanospheres (Ag_60_ and Au_60_) were functionalized with the SERS tag—4-nitrotiophenol (NTP) and PEG via S-metal bonding and with detection antibody due to covalent bonding between the amine group of the antibody and the carboxyl group of PEG (Fig. 1). To immobilise the capture antibody on the gold or silver solid substrate, its surface was modified with (1) 4-mercaptobenzoic acid (MBA) or with (2) the mixture of 11-mercaptoundecanoic acid (MUA) and 11-mercapto-1-undecanol (MU) followed by activation of the carboxylic acid group. As MBA plays a double role of the linker and Raman reporter, its Raman bands can be easily observed in the SERS spectrum in the range of 600–1700 cm^−1^ (Fig. S2 in ESM, black), whereas the thiol reagents (MUA/MU) are not observable in SERS spectrum (Fig. S2 in ESM, red). MUA covalently binds the antigen after NHS–EDC reaction while MU adsorbs naturally between the MUA molecules covering the entire metal surface and preventing non-specific interactions [26]. In consequence, we expected that the SERS signal detecting IL-6 is gathered from both SERS tags, NTP and MBA in the design (1) and NTP only in the assay (2). The Raman reporters’ molecules were strategically selected to provide unique and strong SERS spectra. Taking into account all combinations of the proposed sandwich systems, we examined eight designs as summarised in Fig. 2.

## Characterisation of metal NPs and capture substrates in SERS immunoassays

UV–Vis electronic spectra of metal nanoparticles and nanostructures revealed the presence of localised surface plasmon resonance (LSPR) which plays a fundamental role in generation of surface enhancement of Raman signal. In the case of bare Au nanospheres (Fig S3 in ESM, dark grey trace), a LSPR band was found at 536 nm and was slightly up-shifted after attaching the Raman reporter (NTP) and PEG (Fig. S3 in ESM, red and blue traces, respectively). The conjugation of the SERS nanotag with the detecting antibody did not change the position of the main LSPR band. Additionally, the 279-nm electronic absorption appeared, which originated from π-π^*^ transition of the protein, and thus confirming its successful reaction with the functionalised NPs. For silver nanoparticles, the LSPR absorption was observed at 422 nm (Fig. S3 in ESM, green trace) and the electronic profile behaved in a way similar to AuNP (data not shown).

**Fig. 3:**
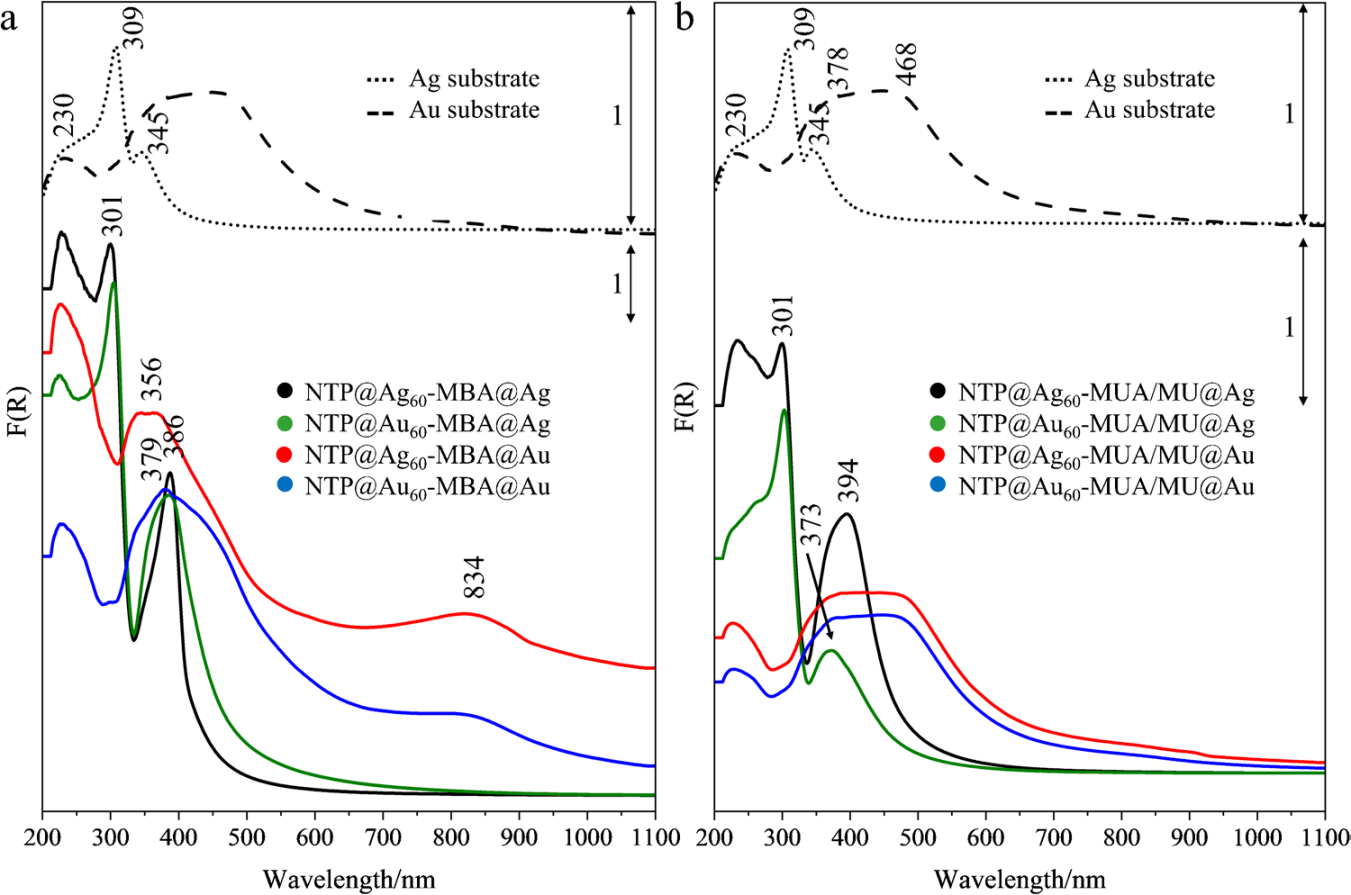
UV–Vis reflectance spectra of the constructed sandwich platforms with two (**a**) and one SERS tags (**b**) compared to the Ag and Au capture substrates

Reflectance extension spectra of the bare Ag solid nanostructures exhibited a well-resolved LSPR signal at 345 nm, whereas covering the AAO template with the Au layer led to the generation of plasmon resonance in a broad region of 300–700 nm (Fig. 3, dotted and dashed traces). Functionalisation of the Au metal with MBA, MUA/MU, and antibody did not affect localised plasmon resonance, whereas in the case of the Ag substrate we observed a significant shift of the LSPR band from 345 to ca. 400 nm (Fig.S4 in ESM). Taking into consideration the overlap of the LSPR positions of both parts of the constructed assemblies, a stronger enhancement of the electromagnetic field was expected for coupling of Ag and Au nanospheres with the Au nanostructured platforms regardless of their functionalisation with the Raman reporter or with the linkers. Figure 3 shows the comparison of the reflectance spectra of the eight sandwich assemblies capturing the IL-6 antigen. All dual-tag sandwich assays completely changed their LSPR characteristics (Fig. 3a). The reflectance spectra are dominated by bands of the capture substrates. LSPR signals of the Ag solid sandwich assays were red-shifted by ca. 70 nm on contrary to the assays with the Au solid substrate. For the latter, the broad band of the bare Au nanoplatform became narrow and blue-shifted to ca. 360 nm. The second LSPR appeared at 834 nm. Differences in the band shape and positions indicated that the coupling between the metal substrates was affected by the metal type of NPs. In the single-tag assay, binding of the IL-6 protein did not change the electronic features of the whole assays when the Au solid substrate was modified with the linkers, suggesting that π-electron system of MBA might contribute through charge-transfer or resonance effect to enhancement efficiency in the dual-tag assays (Fig. 3B). In the case of the Ag capture substrate, LSPR peaks were red-shifted as observed for the dual-tag assays, but higher absorbance appeared for the Ag–Ag than the Au–Ag system.

ATR FTIR spectra collected for both parts of the assays and the entire detection system are displayed in Figure S5 in ESM. Infrared spectra of nanospherical particles functionalized only with NTP and PEG(black trace) as well as with NTP, PEG and the antibody (red trace) showed the presence of bands of NTP at 1105, 1340, and 1510 cm^−1^ and amide I band at 1654 cm^−1^ confirming the successful conjugation of the detection antibody with NPs (FigS5a). Similarly, the functionalisation of the capture part of the sandwich assay was confirmed by FTIR bands of MBA (1084, 1420, 1531, and 1585 cm^−1^), the MUA/MU linkers (1052, 1465, 1737, 2850, and 2918 cm^−1^), and the capture antibody (1653 cm^−1^), see Fig. S5b. Most listed-above IR bands were also observed for the fully conjugated sandwich SERS immunoassays (Fig. S5c).

SERS signals of NTB on Au/Ag NPs were present at 725, 856, 1082, 1338, and 1572 cm^−1^ while a monolayer of MBA deposited on Au/Ag solid substrates generated the 1082 and 1588 cm^−1^ bands (Fig. S6, Table S1 in ESM). Further functionalisation of both metal nanostructures with PEG and antibodies did not affect SERS features of both tags (Fig. S6 in ESM). Since we investigated various metal–metal junctions, SERS spectra of all assay components were acquired with laser excitations at 532 and 633 nm. Surprisingly, capturing of the IL-6 antigen led to quenching SERS signals of the Raman reporters in some combinations, c.f. Table S2 in ESM. Among all investigated cases, the proper readout of the Raman signals was gathered when the Ag and Au solid nanostructured substrates functionalised with MBA and the linkers interacted with the Au nanospheres decorated with NTP upon the 633-nm excitation. This surprising fact cannot be simply explained by the stronger resonance of the LSPR peak and the incident radiation in these cases because we should expect larger surface enhancement under a 532-nm excitation than for the red laser line, see Fig. 3. We also excluded insufficient and/or unsuccessful conjugation of both antibodies with the IL-6 antigen since the same samples were investigated with both laser lines. Thus, we speculated that matching to LSPR peak with the laser excitation does not significantly contribute to the observed signal when the metallic nanostructures interact with each other. A primary reason likely lies in the large surface density of 60-nm gold nanospheres on both planar SERS substrates due to better wetting properties of AuNP and their high biocompatibility for protein immobilisation compared to silver nanoparticles. Thus, both factors positively affected the intensity of the SERS signal that depends on the number of hot spots and tags on both substrates [11, 27].

### Detection of the IL-6 antigen and sensitivity of the sandwich immunocomplex

**Fig. 4:**
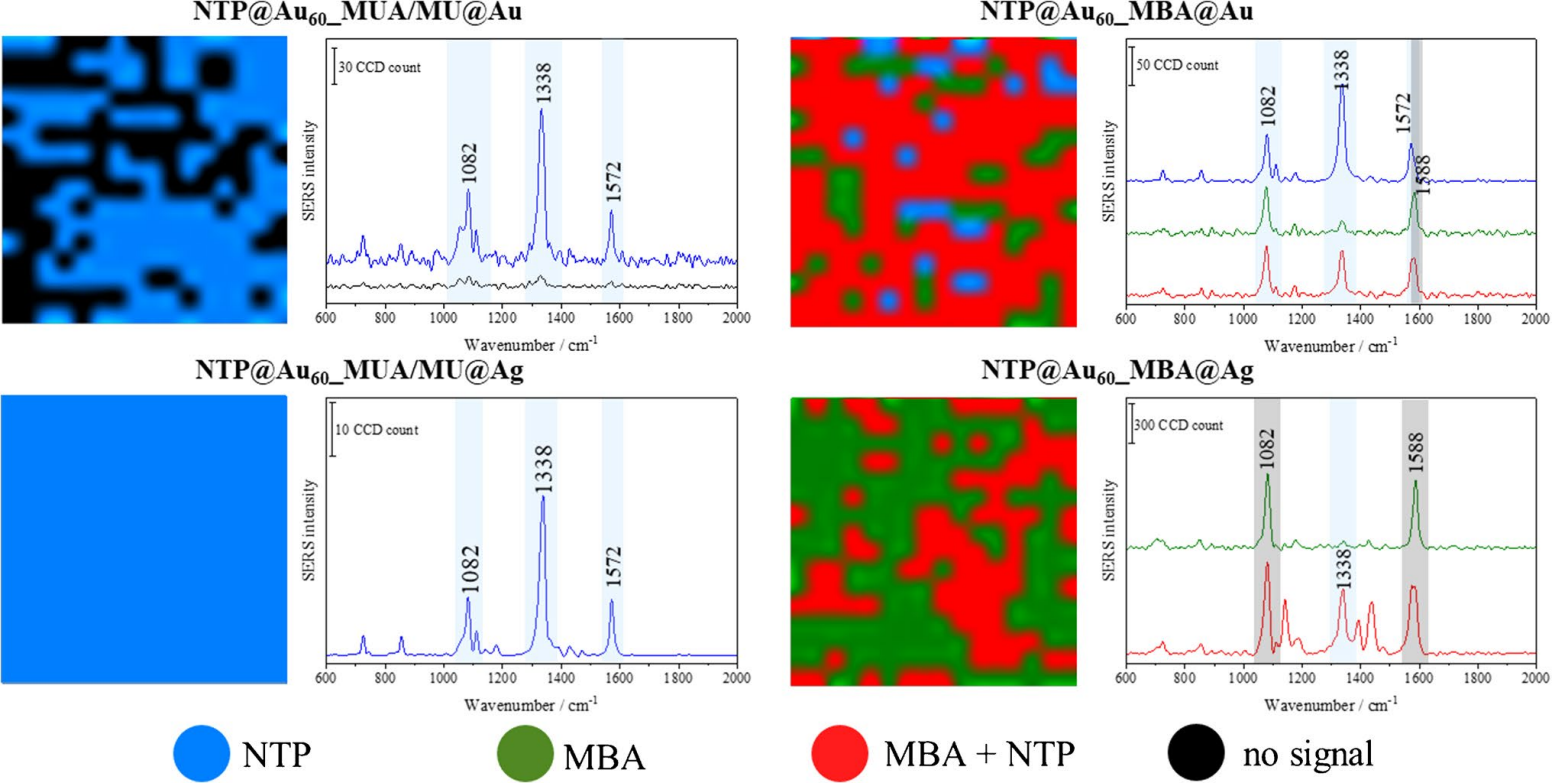
Exemplary KMC false-colour maps of SERS images with their corresponding mean SERS spectra acquired for the sandwich immunoassays constructed from the Au–Au (top) and Au–Ag (bottom) SERS-active substrates. Colour pixels in the maps show the distribution of the SERS signals assigned to NTP only (blue), MBA only (green), and their co-presence (red). In the case of the lack of the SERS signal, pixels are marked in black. The colours in the KMC maps correspond to the colours of the spectra

Based on the above results, we finally investigated the sensitivity of the Au–Au sandwiches in the single- and dual-tag systems for the detection of IL-6. Instead of the collection of SERS spectra from randomly chosen spots on the assay surface, we performed Raman imaging giving in total over 1000 spectra per the assay from a large area of 6400 μm^2^ within 17 min. Such a big number of spectra improves the detection of true-positives and the precision of the signal readout from samples investigated in the wide concentration range of 50–1000 pg mL^-1^. Then, we used *k*-means cluster (KMC) analysis of SERS images to evaluate the signal distribution and to determine the relationship between signal intensity and IL-6 concentrations (Fig. 4). The application of the automatic and unsupervised by the operator chemometric analysis was an added value for detecting true-positives gathered in hyperspectral database improving sensitivity and accuracy of the method.

For the SERS sandwich assays with the capture substrates immobilised with the linkers, only NTP bands were detected (Fig. 4, *left*). The 100% readout of the NTP signal (blue class) was found in the case of the Ag capture substrate, whereas the antigen capturing on the Au nanostructures was efficient up to 60% (Table S3 in ESM). This observation suggests the presence of repulsion forces between Au–Au obstructing successful antibody-antigen–antibody binding if we assume that NTP@Au_60__MUA/MU@Ag is free of the false-positive signal. Averaged SERS spectra of NTP show higher intensity for Au–Ag than for Au–Au assembly (Fig. S7 in ESM left). Considering dual-tag assemblies, we found pixels with true-positives (MBA and NTB, red class), false-positive (NTB, blue class, and MBA, green class), readout (Fig. 4, *right*). More true-positive pixels with higher intensity and reproducibility were observed for the Au–Au (av. 70%) than for the Au–Ag assembly (av. 30%), c.f. Table S3 and Fig. S7, right. Similar to the sandwich assays based on one Raman reporter, in the case of dual-tag system, the better signal was observed for Au–Ag assembly (Fig. S7, right).

**Fig. 5:**
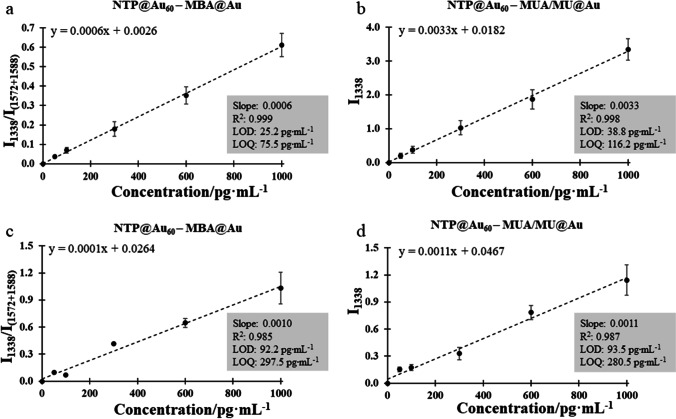
Linear fitting of the peak ratio (1331:1585 cm^−1^) as a function of IL-6 concentrations for the SERS assays built on two (**a**, **b**) and one (**c**, **d**) SERS-active substrates and with dual- (**a, c**) and single-tag labelling (**b, d**)

The analytical performance of the constructed immune assays was determined for Au–Au assemblies in the 0–1000 pg⋅mL^−1^ range of IL-6 concentrations typically observed in biofluids. To evaluate the effect of double enhancement, we determined calibration plots for the corresponding assays prepared on the SERS-inactive Au film. Based on experiments with negative control (BSA instead of IL-6), the contribution of non-specific binding is in the range 9.3–10.1%. A relationship between the SERS signal–IL-6 concentration is plotted for the ratio of two marker bands at 1338 as well as 1588 cm^−1^ of both Raman reporters. The obtained calibration graphs are presented in Fig. 5. All sandwich systems showed very good linearity of the SERS response to concentrations as evidenced by the coefficient of determination value (*R*^2^) in the range of 0.98 and 0.99. The limit of detection (LOD) for two SERS-active plasmonic metals was improved for the dual-tag assembly (25.2 pg⋅mL^−1^) compared to the corresponding assay with the single-tag (38.8 pg⋅mL^−1^), see Fig. 5a, b. The use of the Au hexagonal array instead of the smooth Au film significantly increased LOD from ca. 90 to 25 pg⋅mL^−1^ (Fig. 5a, c). Interestingly, the sandwich assays with the Au film showed similar sensitivity regardless of the number of the tags and LOD values were found to be similar to those reported elsewhere (Fig. 5c, d) [20].

The limit of detection of the pro-inflammatory agent—IL-6 achieved for the optimal combination of the metal–metal junction in the SERS sandwich was 25* pg*⋅mL^−1^. A protocol of this detection method is presented in ESM. In the case of an immunosensor working with the double SERS readout but with the single plasmonic nanostructure (60 nm Au nanospheres like in our case), another inflammatory marker—human α-thrombin was quantified with a much higher LOD of 3.2* ng*⋅mL^−1^ [20]. This comparison indicates that the conjugation of two SERS-active capture and detection substrates significantly improves the detection ability of the SERS-based sandwich assay. Undoubtedly, the incorporation of multi-shaped detection SERS tags (e.g., nanoshells) should offer brighter surface-enhanced Raman signal than nanospheres. Indeed, a few works demonstrated the improvement of the IL-6 detection level up to ca. 4 pg⋅mL^−1^ in microfluidic and magnetic devices [19, 30–32]. From the other side, our results cannot be directly compared with these works because of different analytical parameters and methods used, e.g., lowest detectable concentration instead of LOD, signal-to-noise (S/N) method, without excluding the false-negative signal. We summarised this comparison in Table S4 in ESM.

## Conclusion

The influence of the metal, the number of SERS substrates and tags on the sensitivity of the sandwich-type SERS immune-assay was investigated. The antibody-conjugated NTP-labelled Au and Ag NPs were served as the detection SERS probes. To enhance the signal, the sandwich incorporated nanostructured Au and Ag exhibiting SERS activity and immobilised with the capture antibody. The latter was conjugated through the second SERS tag to improve the readout of true IL-6 antigen binding events. The most binding events with the dual readout of the SERS signal were observed for gold NPs and gold solid substrate. The proof-of-concept for the designed sensor showed that the simultaneous binding of the target protein by two SERS labels in a hot spot between metallic nanostructures detects physiologically relevant target concentrations (over 40 pg⋅mL^−1^). This 3D architecture improved the limit of detection over three times compared to the single-enhancement and -tag assays. The use of the simple nanospherical SERS detection tags and hexagonal metal nanostructures as the capture substrate provided LOD higher than for commercially available ELISA tests quantifying IL-6 (from few pg⋅mL^−1^ to 500 pg⋅mL^−1^) [31–33]. But the latter additionally employed tyramide signal amplification (TSA) improving the level of the quantification. Certainly, a higher enhancement capacity of the SERS immunoassay can be increased by introducing metal nanoparticles and solid substrates of different architectures to manipulate gap distance between them together with maintaining signal reproducibility. Both factors will improve the sensitivity of the immunoassay-based detection platform. Because such an assay has the potential to be used as a PoC (point-of-care) testing tool, further steps should be undertaken to optimise the size of the assay suitable for a portable Raman instrument with preservation of linear relationship between the dual readout and concentration. It will be also challenging to maintain the stability of the SERS tags for the commercial use. This could be possible by using stabilising and life-extending compounds as well as optimising storage conditions before performing tests. Since the proposed SERS immunoassay operates in a way similar to the commonly used ELISA, its application by the biomedical community should be easily translated.

## Supplementary Information

Below is the link to the electronic supplementary material.

Supplementary file1 (PDF 1.33 MB)

## References

[1] Darwish IA (2006). Immunoassay methods and their applications in pharmaceutical analysis: basic methodology and recent advances. Int J Biomed Sci.

[2] Wild D (2013). The immunoassay handbook.

[3] Diamandis EP, Christopoulos TK (1996). Immunoassay.

[4] Grubisha DS, Lipert RJ, Park HY (2003). Femtomolar detection of prostate-specific antigen: an immunoassay based on surface-enhanced Raman scattering and immunogold labels. Anal Chem.

[5] Smolsky J, Kaur S, Hayashi C, et al (2017) Surface-enhanced raman scattering-based immunoassay technologies for detection of disease biomarkers. Biosensors 7: 10.3390/bios7010007 10.3390/bios7010007 PMC5371780 28085088

[6] He J, Fourth E (2013). Chapter 5.1 - Practical guide to ELISA development. Wild DBT-TIH.

[7] Porter MD, Lipert RJ, Siperko LM (2008). SERS as a bioassay platform: fundamentals, design, and applications. Chem Soc Rev.

[8] Wiercigroch E, Stepula E, Mateuszuk L (2019). ImmunoSERS microscopy for the detection of smooth muscle cells in atherosclerotic plaques. Biosens Bioelectron.

[9] Wang G, Lipert RJ, Jain M (2011). Detection of the potential pancreatic cancer marker MUC4 in serum using surface-enhanced Raman scattering. Anal Chem.

[10] Li M, Cushing SK, Zhang J (2013). Three-dimensional hierarchical plasmonic nano-architecture enhanced surface-enhanced Raman scattering immunosensor for cancer biomarker detection in blood plasma. ACS Nano.

[11] Song C, Chen J, Zhao Y, Wang L (2014). Gold-modified silver nanorod arrays for SERS-based immunoassays with improved sensitivity. J Mater Chem B.

[12] Yoon KJ, Seo HK, Hwang H (2010). Bioanalytical application of SERS immunoassay for detection of prostate-specific antigen. Bull Korean Chem Soc.

[13] Granger JH, Granger MC, Firpo MA (2013). Toward development of a surface-enhanced Raman scattering (SERS)-based cancer diagnostic immunoassay panel. Analyst.

[14] Yakes BJ, Lipert RJ, Bannantine JP, Porter MD (2008). Detection of Mycobacterium avium subsp paratuberculosis by a sonicate immunoassay based on surface-enhanced Raman scattering. Clin Vaccine Immunol.

[15] Driskell JD, Kwarta KM, Lipert RJ (2005). Low-level detection of viral pathogens by a surface-enhanced Raman scattering based immunoassay. Anal Chem.

[16] Shin MH, Hong W, Sa Y (2014). Multiple detection of proteins by SERS-based immunoassay with core shell magnetic gold nanoparticles. Vib Spectrosc.

[17] Karn-Orachai K, Sakamoto K, Laocharoensuk R (2017). SERS-based immunoassay on 2D-arrays of Au@Ag core-shell nanoparticles: influence of the sizes of the SERS probe and sandwich immunocomplex on the sensitivity. RSC Adv.

[18] Kamińska A, Witkowska E, Winkler K (2015). Detection of hepatitis B virus antigen from human blood: SERS immunoassay in a microfluidic system. Biosens Bioelectron.

[19] Kamińska A, Winkler K, Kowalska A (2017). SERS-based immunoassay in a microfluidic system for the multiplexed recognition of interleukins from blood plasma: towards picogram detection. Sci Rep.

[20] Chuong TT, Pallaoro A, Chaves CA (2017). Dual-reporter SERS-based biomolecular assay with reduced false-positive signals. Proc Natl Acad Sci U S A.

[21] Scheller J, Chalaris A, Schmidt-Arras D, Rose-John S (2011). The pro- and anti-inflammatory properties of the cytokine interleukin-6. Biochim Biophys Acta - Mol Cell Res.

[22] Qu D, Liu J, Lau CW, Huang Y (2014). IL-6 in diabetes and cardiovascular complications. Br J Pharmacol.

[23] Malek K, Brzózka A, Rygula A, Sulka GD (2014). SERS imaging of silver coated nanostructured Al and Al2O3 substrates. the effect of nanostructure. J Raman Spectrosc.

[24] Hawe A, Hulse WL, Jiskoot W, Forbes RT (2011). Taylor dispersion analysis compared to dynamic light scattering for the size analysis of therapeutic peptides and proteins and their aggregates. Pharm Res.

[25] Hinterwirth H, Kappel S, Waitz T (2013). Quantifying thiol ligand density of self-assembled monolayers on gold nanoparticles by inductively coupled plasma-mass spectrometry. ACS Nano.

[26] Yüce M, Kurt H (2017). How to make nanobiosensors: surface modification and characterisation of nanomaterials for biosensing applications. RSC Adv.

[27] Lin CC, Yang YM, Chen YF (2008). A new protein A assay based on Raman reporter labeled immunogold nanoparticles. Biosens Bioelectron.

[28] Wang Y, Salehi M, Schütz M (2013). Microspectroscopic SERS detection of interleukin-6 with rationally designed gold/silver nanoshells. Analyst.

[29] Wang X, Ma L, Sun S (2021). Rapid, highly sensitive and quantitative detection of interleukin 6 based on SERS magnetic immunoassay. Anal Methods.

[30] Wang X, Ma L, Hu C, et al (2021) Simultaneous quantitative detection of IL-6 and PCT using SERS magnetic immunoassay with sandwich structure. Nanotechnology 32:. 10.1088/1361-6528/abee48 10.1088/1361-6528/abee48 33711835

[31] Human IL-6 ELISA kit. https://www.elabscience.com. Accessed 15 Sept 2021

[32] Human IL-6 ELISA kit. https://www.rndsystems.com. Accessed 15 Sept 2021

[33] ELISA kit for IL-6 detection. https://www.abcam.com. Accessed 15 Sept 2021

