# Supplementary material for: Dual-enhancement and dual-tag design for SERS-based sandwich immunoassays: evaluation of a metal–metal effect in 3D architecture
Source: Mikrochim Acta. 2021 Dec 21;189(1):32. doi: 10.1007/s00604-021-05125-0 (PMC8692285; doi:10.1007/s00604-021-05125-0)
Supplement: Supplementary file 1 — Supplementary file1 (PDF 1.33 MB) [file 604_2021_5125_MOESM1_ESM.pdf]

ELECTRONIC SUPPLEMENTARY MATERIAL

Dual-enhancement and dual-tag design for SERS-based sandwich immunoassays: evaluation of a metal-metal effect in 3D architecture

Ewelina Wiercigroch[1†], Pawel Swit[2†], Agnieszka Brzozka[1], Łukasz Pięta[1], and Kamilla Malek[1,2*]

[1] Faculty of Chemistry, Jagiellonian University in Krakow, Gronostajowa 2, 30-387 Krakow, Poland.

[2] Jagiellonian Centre for Experimental Therapeutics, Jagiellonian University in Krakow, Bobrzynskiego 14, 30-348 Krakow, Poland.

Experimental

Fabrication of metal hexagonal SERS substrates

Highly ordered, gold and silver-coated, porous anodic aluminum oxide (AAO) films at the aluminum foil, were prepared. AAO was fabricated using high-purity aluminum foil (99.999%, Goodfellow (www.goodfellow.com)). Before anodization, Al samples with a dimension of 20×15×0.5 mm were degreased in acetone and ethanol and electropolished in a mixture of $HClO_4$ and C_2H_5OH (1:4 vol.). Electropolishing was conducted under a constant cell voltage of 20 V for 2 min at -3 ºC. The highly ordered porous arrays were formed by two-step anodization of aluminum in 0.3 M sulfuric acid. Anodization of aluminum was performed in a two-electrode cell with Al foil and Pb plate as the anode and cathode, respectively. A constant cell voltage of 25 V and a temperature of 1 ºC were maintained during both steps of anodization. The resulting disordered AAO layer after the first step of anodization was kept in a mixture of 6 wt.% H_3PO_4 and 1.8 wt.% $H_2Cr_2O_4$ at 60 ºC for 1 h. The duration of the first and second steps were 12 and 24 h, respectively. After anodization samples were rinsed thoroughly with deionized water and isopropanol and air-dried. As-prepared AAO/Al substrates were coated with a 5 nm Au or Ag layer by sputter deposition using a vacuum sputter coater (Quorum Q150T S). The sputter coating system was operated under 1×10^{-6} mbar, gold was sputtered under 10 mA for 2 min while silver was sputtered under 25 mA for 2 min.

SEM images (Fig. S1 a and c in SM) confirmed uniform morphology with well-ordered nanopore structures. EDS analysis showed the presence of Ag or Au on the surface of substrates (Fig. S1 b and d in SM) and demonstrated a high and homogenous coverage of substrates by the metal. More detail about morphology and SERS features were reported elsewhere [24].

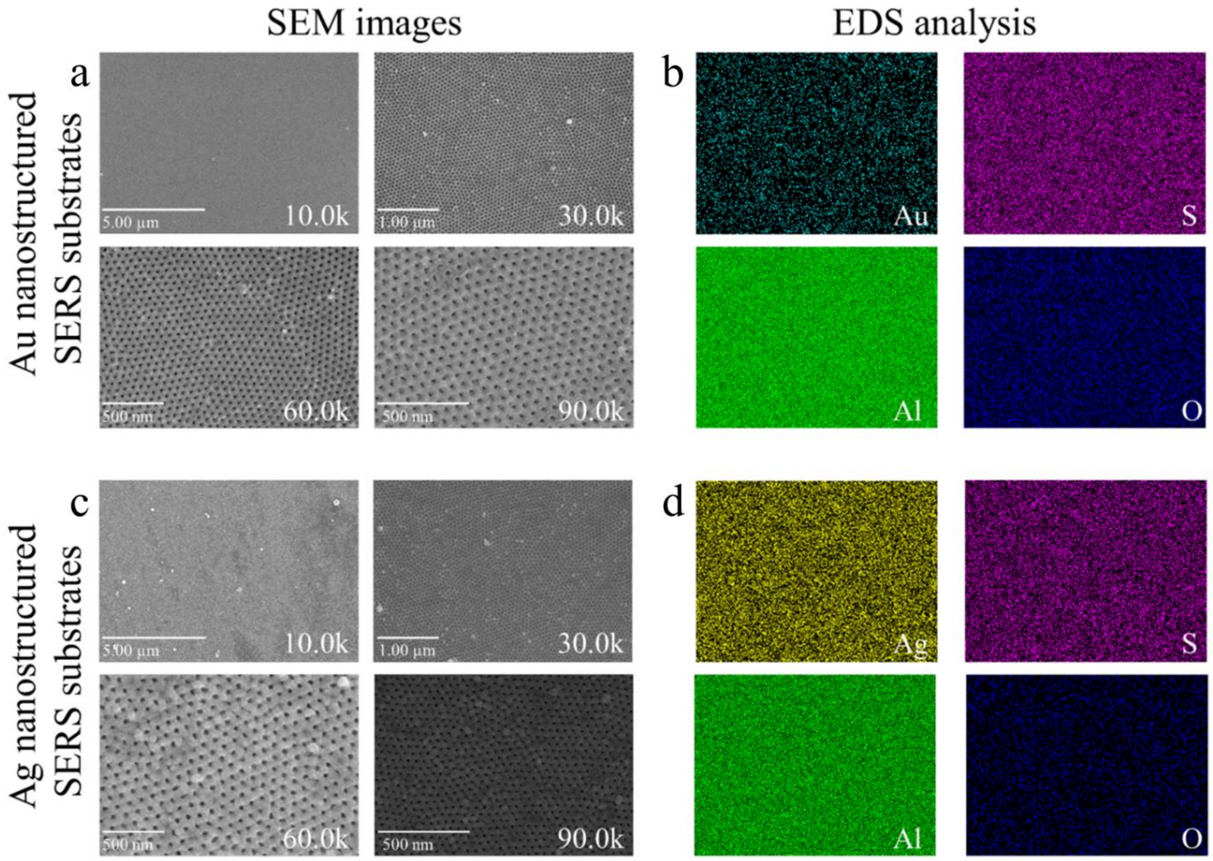

Figure S1. SEM images **(a, c)** and EDS maps **(b, d)** of the Au and Ag hexagonal nanostructures. The cell diameter (Dc) and pore diameter (Dp) of the nanostructured substrates were 56 and 25 nm, respectively, while a double wall thickness (Dw) was calculated to be 31 nm. The porosity of these capture substrates was 18%.

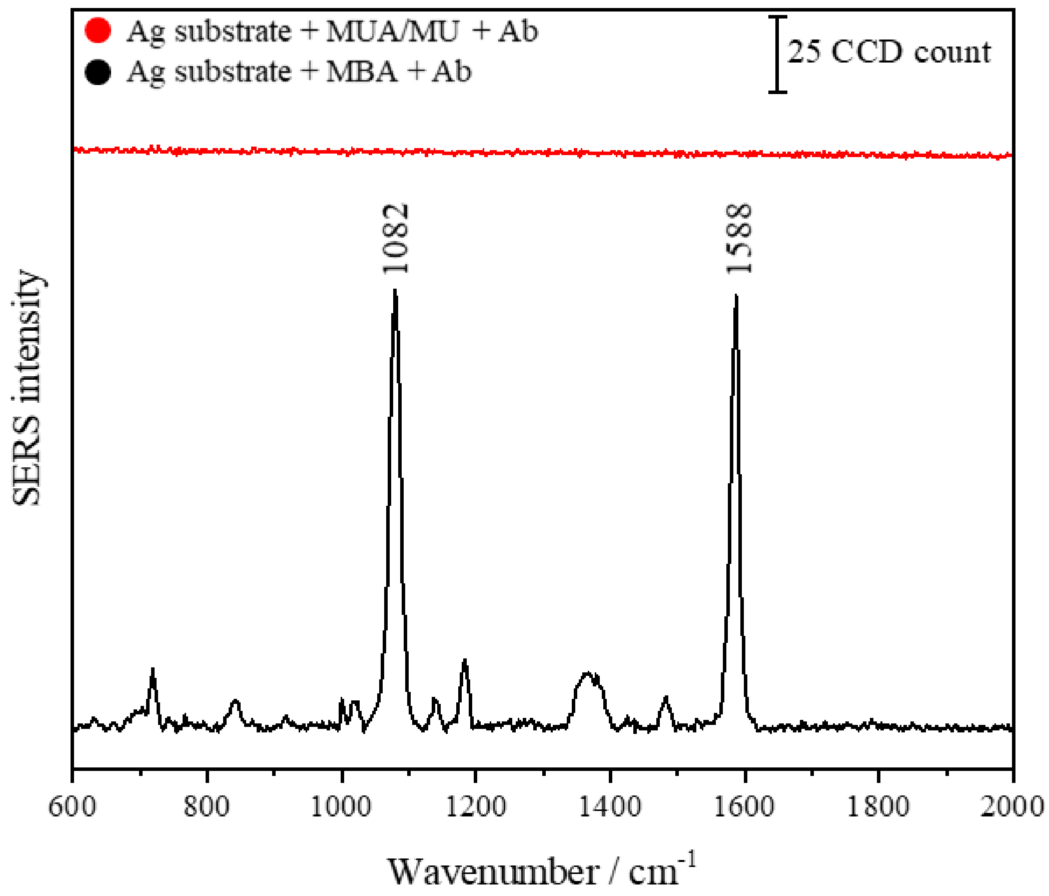

Figure S2. SERS spectra of Ag solid substrates functionalised with MBA and the antibody (Ab) (black) and the MUA/MU linkers and the antibody (red).

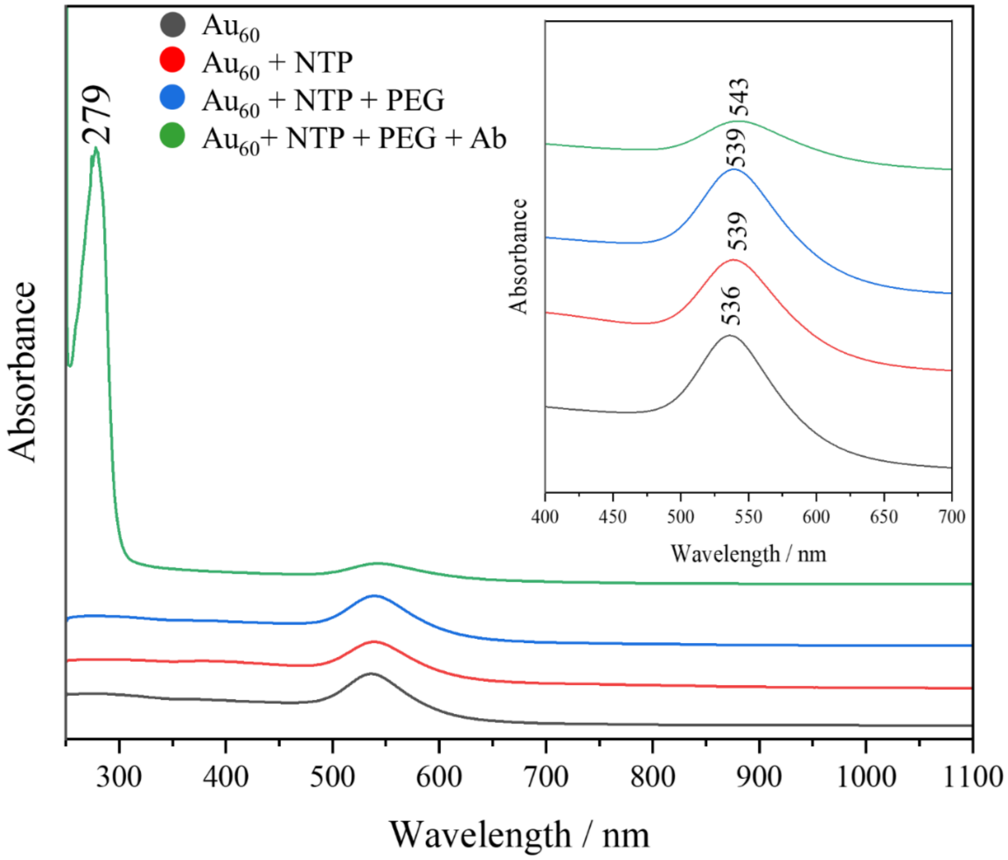

Figure S3. UV-Vis extinction spectra of bare spherical AuNPs with a diameter of 60 nm (black) and after the consecutive functionalisation with the Raman reporter (RR: NTP; red), PEG (SH-PEG-COOH) (blue), and the conjugation with the detection antibody (Ab) (green).

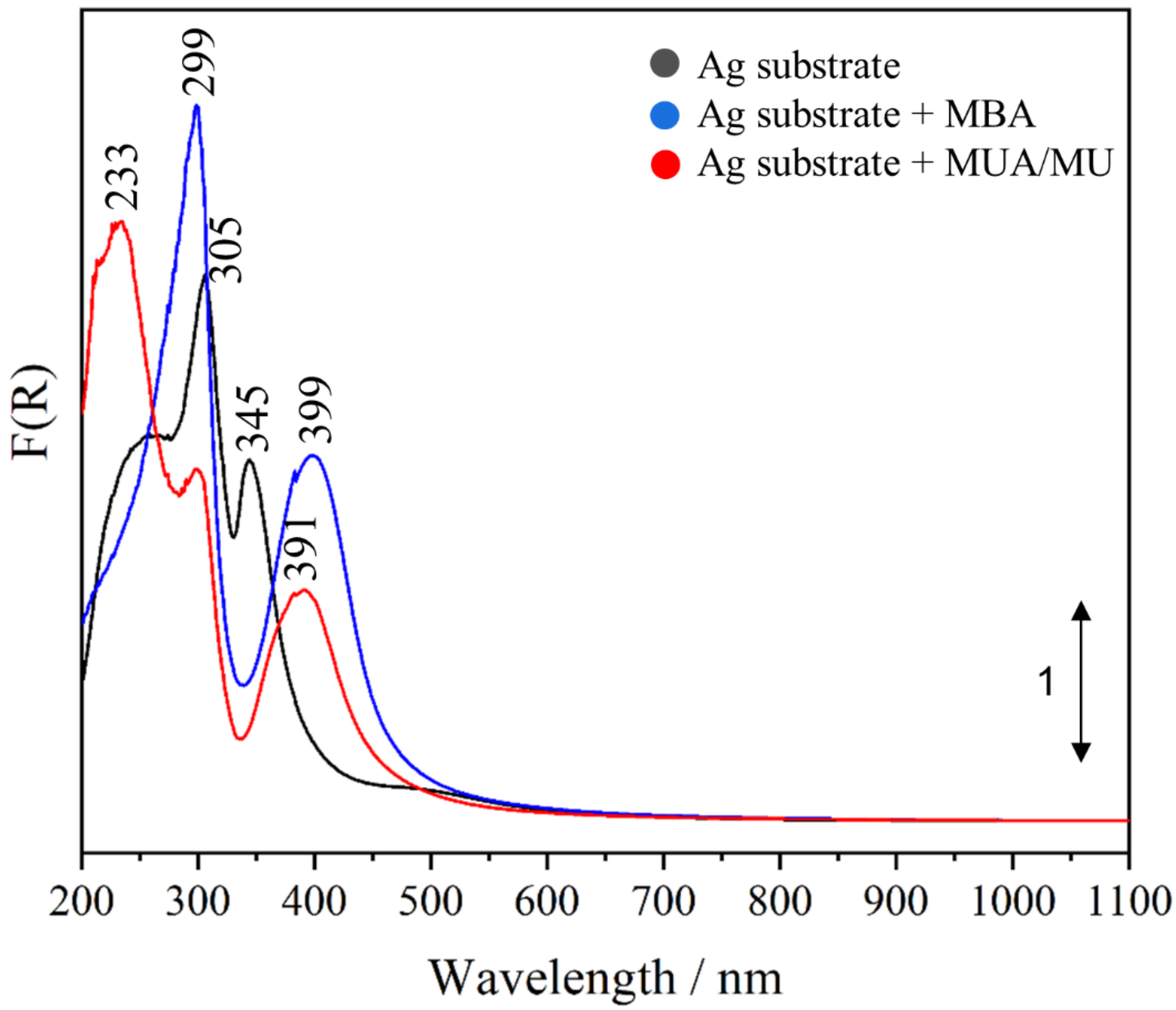

Figure S4. UV-Vis reflectance spectra of Ag hexagonal SERS substrate (black) before and after functionalisation with the Raman reporter (MBA, blue) and the MUA/MU linkers (black, 3x enlarged).

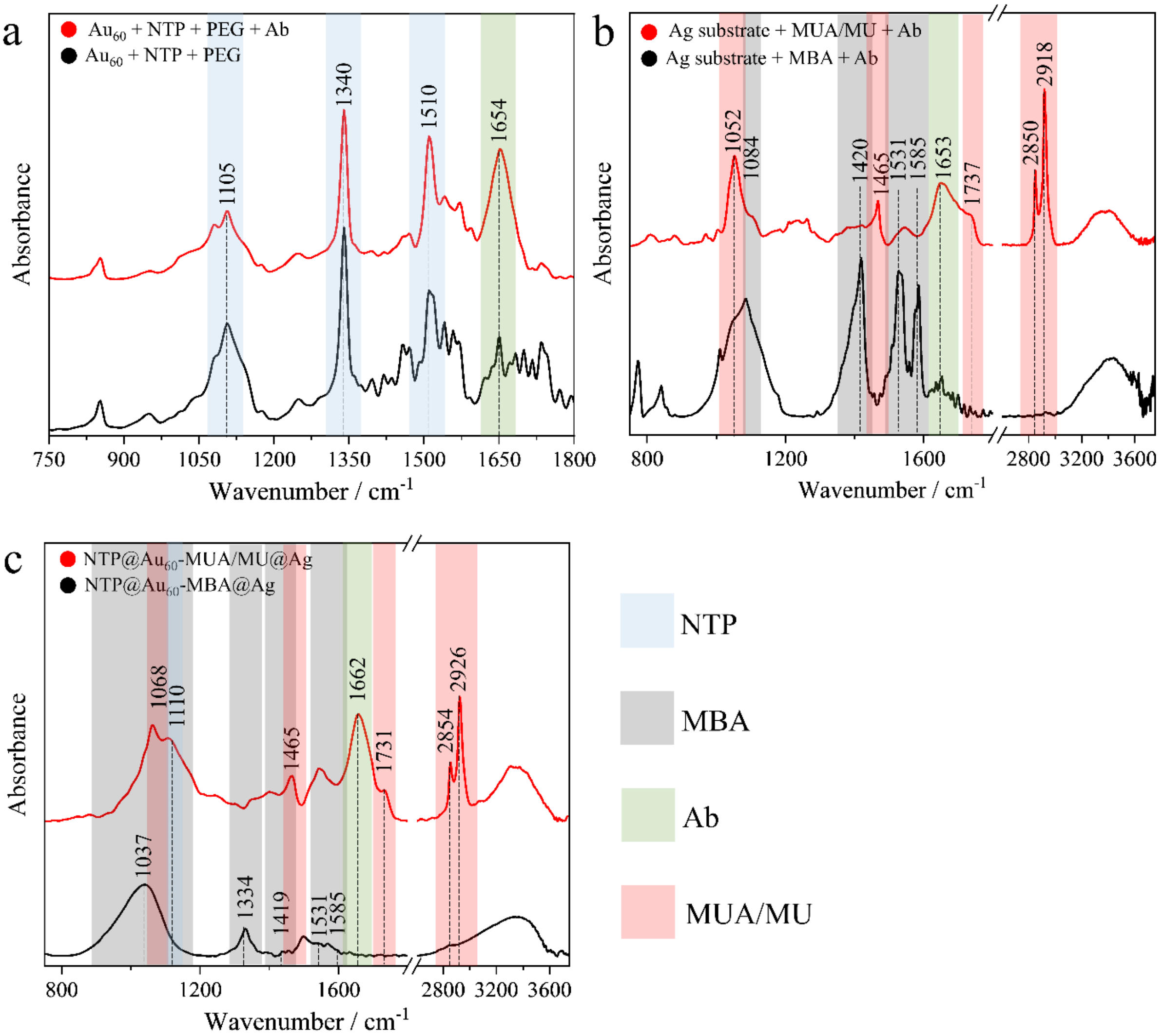

Figure S5. Averaged ATR FTIR spectra of Au NPs functionalized with NTP and PEG (black) and next conjugated with the antibody (Ab, red) **(a)**, Ag capture substrates functionalised with MBA and the antibody (black) and with the MUA/MU linkers and the antibody (red) **(b)**, and the sandwich assays in that the capture substrates were incubated with MBA and MUA/MU **(c)**. Shadings denote marker IR bands of the components.

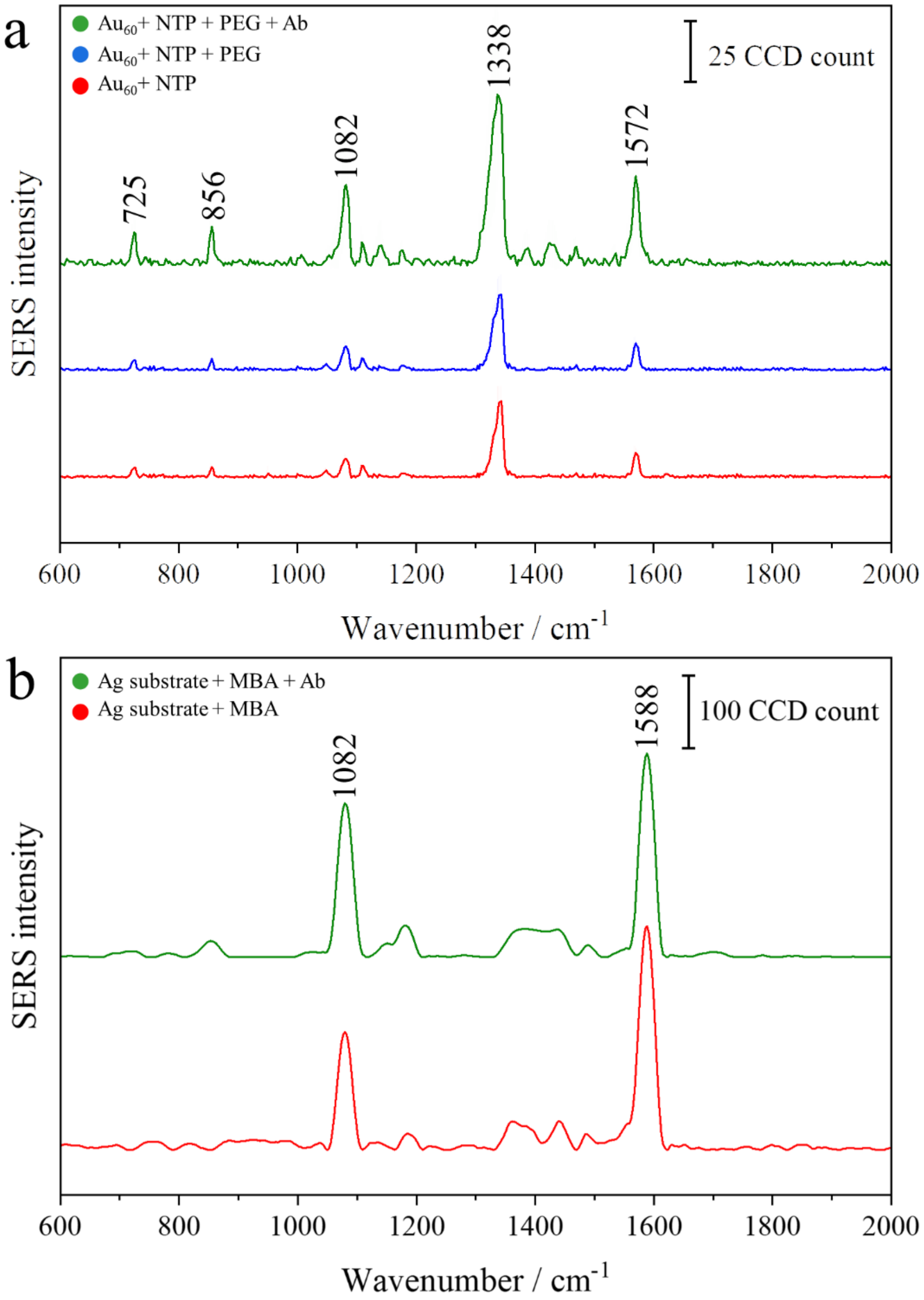

Figure S6. SERS spectra from the consecutive functionalisation steps of the sandwich parts: **(a)** for AuNPs with NTP (red), with NTP and PEG (blue) and with NTP, PEG, and the antibody (green); **(b)** for Ag solid substrate incubated with MBA (red) and MBA and the antibody (green).

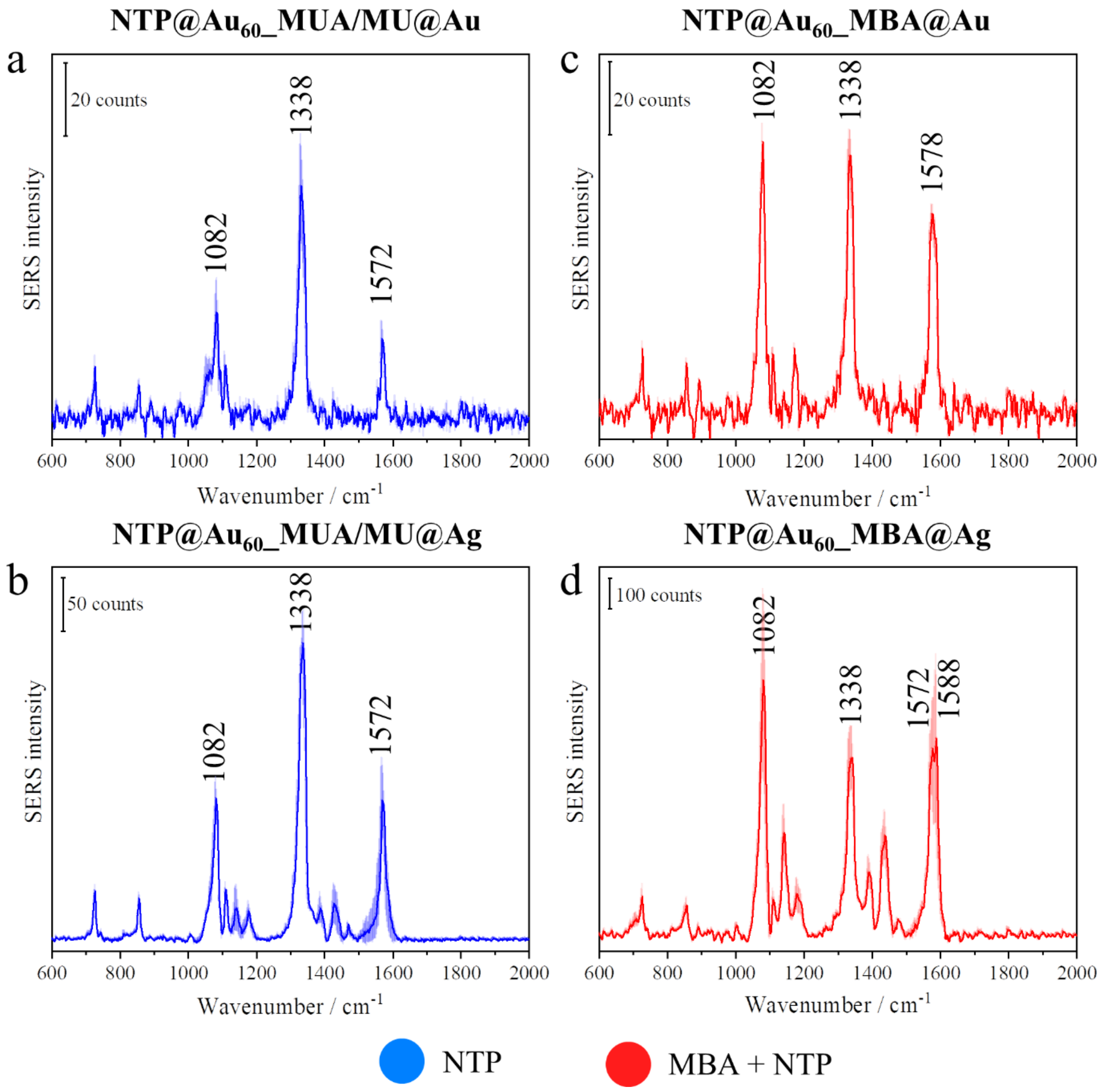

Figure S7. Averaged SERS spectra of the sandwich assay: **(a)** NTP@Au60_MUA/MU@Au, **(b)** NTP@Ag60_MUA/MU@Ag, **(c)** NTP@Au60_MBA@Au and **(d)** NTP@Au60_MBA@Ag, showing SERS bands of NTB **(a, b)** and MBA and NTP **(c, d)**.

Table S1. SERS bands observed in the capture and detection substrates separately as well as in the sandwich assays.

**Assembly**		**SERS bands / cm^{-1}**
Au_{60} + NTP		725, 856, 1082, 1338, 1572
Au_{60} + NTP + PEG		
Au_{60} + NTP + PEG + Ab		
Ag_{60} + NTP		1080, 1346, 1574
Ag_{60} + NTP + PEG		1080, 1340, 1574
Ag_{60} + NTP + PEG + Ab		1080, 1338, 1572
Ag/Au substrate + MBA		1082, 1588
Ag/Au substrate + MBA + Ab		
Ag/Au substrate + MUA/MU		Not observable
Ag/Au substrate + MUA/MU + Ab		
NTP@Au_{60}_MUA/MU@Au	NTP	1082, 1338, 1572
NTP@Au_{60}_MUA/MU@Ag	NTP	1082, 1338, 1572
NTP@Au_{60}_MBA@Au	NTP	1082, 1338, 1572
	MBA	1082, 1588
	MBA+NTP	1082, 1338, 1578
NTP@Au_{60}_MBA@Ag	MBA	1082, 1588
	MBA+NTP	1082, 1338, 1588

Table S2. A summary of SERS readout in the sandwich immunoassays irradiated with laser excitations at 532 and 633 nm (bold assemblies denote a proper signal observed in SERS spectra).

**Assembly**	**532 nm**	**633 nm**
**NTP@Au$_{60}$_MBA@Ag**	MBA only	**NTP and MBA**
NTP@Ag$_{60}$_MBA@Ag	MBA only	no signal
**NTP@Au$_{60}$_MBA@Au**	no signal	**NTP and MBA**
NTP@Ag$_{60}$_MBA@Au	no signal	MBA only
**NTP@Au$_{60}$_MUA/MU@Ag**	no signal	**NTP**
**NTP@Ag$_{60}$_MUA/MU@Ag**	**NTP**	no signal
**NTP@Au$_{60}$_MUA/MU@Au**	no signal	**NTP**
**NTP@Ag$_{60}$_MUA/MU@Au**	no signal	**NTP**

Table S3. Pixel distribution of the SERS signals of the Raman reporters (in %, ±SD) observed in images of the Au-Au immunoassays. Calculated from 4 images consisting of 1024 pixels in total per the sandwich assembly.

**Assembly**	**Coverage [%]**		
	**NTP**	**MBA**	**MBA + NTP**
**NTP@Au$_{60}$_MUA/MU@Au**	51 ± 9	-	-
**NTP@Au$_{60}$_MUA/MU@Ag**	100 ± 0	-	-
**NTP@Au$_{60}$_MBA@Au**	22 ± 17	16 ± 3	68 ± 21
**NTP@Au$_{60}$_MBA@Ag**	-	69 ± 11	31 ± 11

Table S4. An overview on recently reported nanomaterial-based SERS methods for the determination of IL-6.

Materials used	Figures of merit						Samples	Dual-tag	Ref.
	LOD	Linear range	R^2	Sensitivity	Precision	Accuracy			
**rationally designed gold/silver nanoshells (Au/Ag-NS)**	1 pg/mL*	1 pg/mL to 1 µg/mL	-	high	-	-	synthetic	-	28
**bimetallic Ag-Au sandwich immunoassay in microfluidics techniques**	3.8 pg/mL	0–30 ng/mL	0.9919	high	<8% (RSD)	-	blood plasma	-	19
**SERS magnetic immunoassay: core–shell nanoparticles and magnetic beads**	1.6 pg/mL	0–1000 pg/mL	0.9997	high	<15% (CV)	93.9-99.1% (recovery)	serum	-	29
**SERS magnetic immunoassay: core–shell nanoparticles and magnetic beads**	0.54 pg/mL	0–1000 pg/mL	0.9985	high	<20% (CV)	89.8- 104.2% (recovery)	serum	-	30
**dual-SERS tag sandwich assays Au-Au**	25.2 pg/mL	0–1000 pg/mL	0.999	high	<10% (RSD)	-	IL-6 in buffer, not established on real sample	+	present work

* lowest detectable concentration (LDC)

IL-6 SERS-based sandwich immunoassay Kit

For the quantitative measurement of IL-6 in biological sample.

Materials Supplied

Item	Quantity	Storage Condition
Au solid SERS active substrate (10 mm x 10 mm)	30*	+4ºC
Au nanospherical SERS-active particles	2 mL	+4ºC
0.1 mg·mL^{-1} Interleukin-6	10 µL	+4ºC
0.5% Tween-20 in PBS	100 mL	+4ºC
Sample diluent NS	50 mL	+4ºC
PBS	250 mL	+4ºC

* 7 for the calibration procedure

Reagent Preparation

0.05% Tween-20 in PBS: Prepare 0.05% Tween-20 by diluting obtained solution (0.5% Tween-20) ten times with PBS.

Standard Preparation

Always prepare a fresh set of standards for every use. The following section describes the preparation of a standard curve for triple measurements (recommended).

1. Label eight tubes, 1 for a working solution, and 7 for the standard solution
2. Prepare working solution (WS) of IL-6 at conc. 100 ng·mL^{-1} by adding 1 µL of IL-6 (0.1 mg·mL^{-1}) to 999 µL PBS. Hold at room temperature and mix gently
3. Use the working solution to prepare the following dilution series. Standard with conc. 0 pg·mL^{-1} contains no protein and is the blank control.

IL-6 conc. (pg·mL^{-1})	Volume of IL-6 at given conc.	Volume of PBS
**1000**	10 µL (100 ng·mL^{-1})	990 µL
**600**	6 µL (100 ng·mL^{-1})	994 µL
**300**	3 µL (100 ng·mL^{-1})	997 µL
**100**	300 µL (300 pg·mL^{-1})	600 µL
**50**	300 µL (100 pg·mL^{-1})	300 µL
**25**	300 µL (50 pg·mL^{-1})	300 µL
**0**	0 µL	1000 µL

Sample Preparation

Serum: Samples should be collected into a serum separator tube. After clot formation, centrifuge samples at 2,000 x g for 10 minutes and collect serum. Dilute samples into Sample Diluent NS and assay. Store un-diluted serum at -20ºC or below. Avoid repeated freeze-thaw cycles.

Time: 20 min.

Cell Culture Supernatants: Centrifuge cell culture media at 2,000 x g for 10 minutes to remove debris. Collect supernatants and assay or dilute samples into Sample Diluent NS and assay. Store un-diluted samples at -20°C or below. Avoid repeated freeze-thaw cycles.

Time: 20 min.

Assay Procedure

1. Apply 200 µl of previously prepared sample and standards of IL-6 solution to the Au solid SERS active substrates (10 mm x 10 mm) placed in a resealable box.

2. Incubate for 1 h at room temperature.

3. Add the freshly prepared 0.05% Tween-20 in PBS in small crystallizers up to a height of 3 mm. Rinse by dipping the substrates 3 times in fresh aliquots of the solution. After rinsing, place the substrates in the box.

4. Apply 200 µl of Au nanospherical SERS-active particles to the substrates.

5. Incubate for 1 h at room temperature.

6. Add the freshly prepared 0.05% Tween-20 in PBS in small crystallizers up to a height of 3 mm. Rinse by dipping the substrates 3 times in fresh aliquots of the solution. After rinsing, place the substrates in the box.

7. Allow the sensors to dry. The sensors prepared in this way are ready for measurements.

Data collection and analysis

1. Collect Raman spectra:
a) excitation wavelength: 633 nm
b) laser power: ca. 0.5 mW
c) objective: at least 40x
d) No of spectra: at least 50
2. It is recommended to do at least 2 repetitions.
3. Calculate intensity ratio of NTB and MBA bands, determine the calibration plot and calculate the concentration of the analyte.
